# Editorial: The Psychology of Food Safety and Consumption

**DOI:** 10.3389/fpsyg.2021.767212

**Published:** 2021-11-18

**Authors:** Fu-Sheng Tsai, Xiao-Wei Wen, Shalini Srivastava

**Affiliations:** ^1^North China University of Water Resources and Electric Power, Zhengzhou, China; ^2^Department of Business Administration, Cheng Shiu University, Kaohsiung, Taiwan; ^3^Center for Environmental Toxin and Emerging-Contaminant Research, Cheng Shiu University, Kaohsiung, Taiwan; ^4^Super Micro Mass Research and Technology Center, Cheng Shiu University, Kaohsiung, Taiwan; ^5^College of Business, South China Agricultural University, Guangzhou, China; ^6^Jaipuria Institute of Management, Noida, India

**Keywords:** consumer psychology, food consumption, food safety, governance, cognition and behavior

## Introduction

Food safety and food security are both central issues for human welfare and well-being in modern society. The importance of these two issues has led global leaders to invest their efforts and capital heavily in improving food quality and governance. On the other hand, these issues as phenomena are especially critical when embodied in the context of food consumption. Issues regarding food safety, security, and consumption are greatly connected through psychological mechanisms. After all, it is the consumers' inner psychological and cognitive functions of food safety that may directly determine their intentions and behaviors toward food consumption. What might be equally important but long-neglected by empirical studies is the reverse logic that food consumption *per se* may alter consumers' psychological interpretation of food safety.

For either case, more innovative studies are required to advance our current knowledge, and to build a formal research stream. For example, trust in safety has long been examined as a vital factor in affecting food consumption, while the issues of “how,” “when,” and “why” daily or specific food consumption experiences may influence consumer's long-term trust in safety (and trust in “whom”) have not been explored systematically. In such veins, further academic works are desired, in the topics such as the following: new psychological mechanism(s) exploration for food safety and consumption; research methodology and analytic approaches; contexts-specific (e.g., online food shopping) studies; integration with other disciplines (e.g., Sociology, Economics, Politics); comparative studies (e.g., same issues in different cultures or sub-cultures); further outcomes for the psychology of food safety and consumption (e.g., habit); policy- and governance-oriented studies/opinions; green and sustainable food safety and consumption, etc. New thoughts and insights need to be stimulated and generated for food safety and consumption research.

In the published Research Topic, several pioneer studies heeded the call and supported the intended development with their unique contributions. We will review these papers that successfully push the research frontiers of this literature and propose future possibilities in theoretical and practical developments. For integration, we develop a typology with two axes—level of analysis and physical vs. virtual spaces — to locate each of the published research's strategic position in the literature (see Table 1 below).

**Table 1:** A typology of the published articles on food psychology.

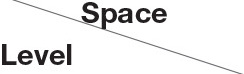	**Physical field**	**Virtual field**
Macro-level	He et al. (cost-sharing strategy in food supply chains) Huang and Lin (food governance as a psychological mechanism for poverty alleviation) Hu et al. (Price policy of raw milk) Chen et al. (Food and environment and green eating)	Liu and Lin (online food shopping)
Meso-level	Liu and Lin (organizational culture and food safety construction) Meng et al. (Consumer behavior → social co-governance) Hu et al. (Global trade networks of dairy food) Nie et al. (balance between food safety control and food quality improvement)	Lin and Wu (food traceability system)Tsai et al. (System attributes of continuance intention toward food safety information from social media)
Micro-level	Wu et al. (comprise effect in consumer choice)Shan et al. (framing and anchoring effects and consumer intention)Ruangkanjanases et al. (green purchase behaviors)Xu et al. (Decoy effect and purchase for welfare animal product)	You et al. (purchase Intention of Organic Food *via* Social Media)

## Research Frontiers And Imagination For Future Studies

Wu et al. tried to uncover the face of the compromise effect in the specific context of consumer choices for food products. Having been well-discovered in general consumer behavior research, the compromise effect has not yet been examined well in the food consumption circumstance. The researchers investigated pork purchase decisions of consumers based on the setting of different decoy information, alone with consumer health-related factors considered together. As a result, they found that “… consumers exhibit significant compromise effects after receiving both low-price and high-price decoy information. However, when decoy information is presented after consumers have made choices without decoy information, their behavior changes systematically with a weakened compromise effect.” The study's contributions are 2-fold: it not only contributed to the traditional consumer choice behavior research by extending its applications to food purchase context, but it also contributed to food safety and traceability studies by adding knowledge with the incorporation of the mature construct of compromise effect.

Ruangkanjanases et al. look deeply into consumers' green (organic) food purchase intention. A group of determinants (i.e., individual benefits, social benefits, willingness-to-pay, environmental responsibility, e-word-of-mouth, values, self-competence, convenience, and environmental literacy) were tested for their effects on the purchase intention. The results showed that all factors except for the subjective norms are positively influential on purchase intention. The implications for the social responsibility of consumption against the backdrop of “the green wave” in society are seriously discussed. The major contribution of this study is not just limited to the area of food purchase; on the contrary, the widely used and accepted Theory of Planned Behavior is extended by the authors' empirical efforts and conceptual reflections. From the paper, we could also observe that purchase intention for green products is inherently a multilevel, multidisciplinary, but not just psychological, phenomenon beyond ourselves. A complex world in the perception of the purchasers projected the real one and the consistency and/or conflicts between the two worlds implicitly guide the consumers' cognition and behavior consequently.

The knowledge-oriented article by Shan et al. explores consumers' attitudes and purchase intentions toward organic food from the framing and anchoring effects perspective. They found that whether being positive or negative, message framing significantly influences consumers' attitudes and purchase intention. Specifically, “a negatively framed message induces a more favorable attitude and purchase intention than a positively framed message, a low anchor price is more favorable than a high one, and the interaction effect of framing and anchoring is not significant at the 1% level.” Moreover, an anchor price in advertisements alters a consumers' judgment. Finally, being equipped with less organic food knowledge makes consumers more susceptible to the aforementioned framing and anchoring effects. Together, such results clearly called for a delicate framing message strategy, integrating with price anchoring practices in the context of consumer knowledge.

He et al. asked an interesting question: “Whether the traditional revenue sharing or cost-sharing strategy is still efficient in the food supply chain.” This is a typically good example of “old-wine-in-new-bottle” research that could make contextual contributions and add new knowledge. The paper's focus is on green innovation efficiently motivated by traditional cooperation contracts in the food sector. The Stackelberg equilibrium structure is utilized, which also makes a methodological contribution to the food sector studies. The results show that “when the supply is interrupted due to the insufficient stimulation of green consumption at the market demand side, manufacturers need to stimulate their green innovation efforts by sharing the cost of suppliers, and the cost-sharing proportion is affected by the marginal profit coefficient of manufacturers and suppliers.” Such results also influence the marginal profit of suppliers and manufacturers and the overall income of the food supply chain.

You et al. argued that when consumers buy organic products in the market, complete information is lacking, as compared to the traditional channels such as newspapers, magazines, and television advertisements. Against such backdrops of information asymmetry, social media rises and becomes a major informational source for organic foods. The analyses found that task characteristics and technology characteristics had significant effects on consumer expectation confirmation and perceived usefulness through the task-technology fit. Then, confirmed expectations and perceived usefulness, in turn, influenced satisfaction and continuance intention. The results are useful in that the practitioners know better about how to utilize social media as a platform-based strategy for organic food promotion.

In the Tsai et al. study, virtual community websites are depicted as a platform for people with common interests in food safety to extend their social relations and engagement in foods affairs in social media. Based on such premise, the study proposed a model for assessing antecedents of continuance intention toward food safety information from social media. Using Facebook as an example, an integrated model of the expectation-confirmation theory and technology acceptance model with technology readiness as a moderator has been examined. The results showed that “perceived ease-of-use, usefulness, and confirmation indirectly affected social media continuance usage intention through satisfaction; perceived ease-of-use, usefulness, and satisfaction were the direct determinants that affected users' social media continuance intention. Furthermore, positive technology readiness had significant effects on the relationship between perceived ease-of-use, usefulness, confirmation, satisfaction, and continuance intention toward food safety information.” Overall, very rich information has been demonstrated for practical and academic references.

Liu and Lin's conceptual efforts tended to emphasize the fundamental differences between online vs. physical shopping in the food sector. She proposed that “1: The design and implementation of online food shopping (eco)systems should engage the consumers and other stakeholders to co-create collective and social values; 2: A better fit between technologies' and food businesses' natures could generate better applications for online food shopping; 3: A business model with sound finance systems becomes the core of a healthy online food ecosystem; 4: The interaction and transformation between online (virtual) and offline (virtual) food businesses determines the dynamic development of future food shopping.” All of those propositions represent promising opportunities for future empirical examinations.

Meng et al. investigated consumers' agency in food safety social co-governance. The central functionality of active consumers for constructing food safety social co-governance was emphasized and examined. For examinations, the authors developed a multidimensional questionnaire on consumer psychological capital that could be used to measure the degree of consumer participation in food safety social co-governance. Analyses based on multiple samples showed that a 4-factor model with 23 items explained 61.05% of the total variance with reliability and validity were both confirmed. The developed questionnaire can serve as an instrument to follow by future studies to expand psychological capital-related research in the context of food governance.

Liu and Lin put culture and CSR back in the pursuit of high-quality food from an organized human resource perspective. A very frank statement reflected the truth of centering the practices back to people as the micro-foundation for supporting institutions. As they stated, “…No matter how regulations are coercively released and implemented, the free will and behaviors of human actors (e.g., employees) leads to a real result in food safety.” And the solution proposed by the authors was an organizational culture that can mold personnel behaviors and stimulate safety-oriented actions. Almost perfectly, the authors used Walmart as an example to demonstrate interwoven green organizational culture, corporate social responsibility, and food safety. This article is another good example of taking stock of a mature issue and applying it in a significant new context to generate both theoretical and practical implications.

Huang and Lin article reads like one that is eager to resolve the grand challenge of poverty alleviation for all. True as it is, poverty is such a grand challenge and under its influences, food insecurity might be seriously affected (mostly in a negative direction). This article discusses food governance as a psychological mechanism to facilitate the sense of wellness in people's minds in the context of poverty alleviation. Mainly, we argued that when a government is implementing poverty alleviation, not only economic efforts for people's living but also good psychological feelings are required. We thus argued that sound food governance may increase the sense of wellness in the minds of people as food consumers, by increasing food safety and security. This perspective paper contributes by explicating the influences of macro-level governance design in safer and more secure food systems on people's psychological wellness, especially in the background of poverty alleviation in developing countries.

Lin and Wu put their attention toward food traceability systems that serve as an important modern mechanism for facilitating food safety. They tested for the influencing factors on the food traceability system from integrative theories. Push factor (information system success model), pull factor (ITM theory), mooring factor (TPB), and switching intention were integrated into the push-pulling-mooring theory (PPM) to form a useful framework to study the switching intentions of two-dimensional code traceability technology for dairy products. The results of the survey study indicated that the influencing factors of thrust, pull, and mooring force were identified. As consumer choice of traceable safe food is critical for public health and economic consumption, and the integrated multi-model framework that the authors proposed is of practical value in identifying ways to strengthen consumer willingness in using QR code traceable system for food products and to improve consumer confidence in the use of food technology.

Hu et al. offer a clear route in assessing the price policy of raw milk, with the impact of the policy implementation on milk price and profit distribution in the supply chain examined. The results told us that the price of raw milk differs with supportive vs. unimplemented price policies; that the aforementioned differences are obvious when considering regional differences; and that the guidance policy for milk price drives the price increase or price suppression due to an intent for a balanced profit distribution in the supply chain. This study effectively links factors across different levels of analyses (i.e., psychological, organizational, industrial, and institutional) to explore the interlinked and sustainable dynamics between product, price, profit, and policy in the food sector.

Another study from Hu et al. constructed evolutionary network characteristics of the world dairy industries based on the overall trade pattern. Specifically, the evolution of trade blocs and the co-opetition relationships involving dairy products in major economies were compared. The results show that continuous and complex changes have taken place in the world's dairy trade network since 2001, while the number of trade entities in dairy products has stabilized since 2012. Mainly, a small-world effect and scale-free property exist in the world dairy trade network. Also, factors such as geographical positions, historical cultures, and political relations have led to the evolution of the trade blocs in the world. As the study sketched both the structural and social patterns of the trade networks, the results indeed offered a social-psychological foundation for policy-makers when making decisions of international dairy trade.

Chen et al. inform us that the imperatives of safe foods are highly tied to the affairs of a clean environment. As global climate change has become the central issue of all mankind, consumers' will and efforts to both maintain environmental soundness and food quality are affected by consumers' consumption habits (and their changes). The trend of food overconsumption with high food waste calls people to eat “greenly” and so eco-friendly. In such a background, the study examined intrinsic motivations (e.g., social recognition, environmental ethics, joy, etc.) and their interrelationships that influence consumers' green eating intention. The results showed that “social recognition and environmental ethics have significant effects on curiosity, joy of purchase, perceived usefulness, subjective norm, and perceived behavior control” and that “the mediator between environmental ethics and behavior intention are joy of purchase, perceived usefulness, subjective norm, and perceived behavior control.” As to our knowledge, this study is among the first to construct a detailed and complex model to inform subtle factor structures for green eating. The results of this study, hence, can be referred to by other groups of people who also wish to eat in an environmentally friendly way.

Wu et al. looked into consumer preferences for traceable pork attributes based on a system composed of traceability, animal welfare, place of origin, and price attributes. Choice experiments and Bayesian inference analysis were adopted as methods of examination. Results showed that both complementary and substitutive relationships existed between dietary animal welfare and traceability information and also between health welfare and non-indigenous vs. indigenous production. These results are especially informative in the context of global health and food-related crises such as the COVID-19 pandemic. Specifically, the study suggested that “the government should encourage manufacturers to produce diverse traceable animal-derived food not only to protect animal welfare and promote the construction of ecological civilization, but also to develop new animal-derived food markets to satisfy different levels of consumer demand.”

Xu et al. analyzed the relationships between consumer behaviors when purchasing meat products produced with animal welfare under different decoy scenarios. Hundreds of consumers purchasing pork and chicken were observed in four types of decoy scenarios based on breeding time, breeding model, diet cleanliness label, and price attributes. A decoy effect was observed in a bounded rational consumption situation in relation to both chicken and pork purchasing behaviors. Comparing the two types of consumption experiments, price decoy still played a significant role. The results of the experiments suggested strengthening people's knowledge of livestock welfare, designing a breeding model decoy or price decoy in the process of chicken sales, and designing a diet cleanliness label decoy or price decoy in the process of pork sales. Suggesting so, the study helps accurately understand consumer minds and behaviors and reduces biases during meat consumption.

Last but not least, Nie et al. noted that food safety that keeps the consumers safe and food quality that leads to consumer satisfaction must be considered together when studying food management systems. This study examined this by focusing on the influence of loss aversion on one's psychological level and of income effect on one's socio-demographic level. The findings indicated that “…loss aversion and income effect significantly influence the way consumers value food safety vs. quality labels when considering potential health risks and food price. High risk-averse and low-income consumers with strong loss aversion and a weak income effect show a higher demand for food safety labels as a way to ensure easy access to safety indications. Low risk-averse and high-income consumers with weak loss aversion and a strong income effect show a higher demand for food quality labels because they hope to gain more health benefits from high-quality food at good prices.” The contribution of this study is to remind governments, manufacturers, consumers, and all related stakeholders to find a balance between food safety control and food quality improvement when facing diverse market demands and preferences transition.

## Concluding Remarks

Psychology and Food Science are two distinctive but potentially highly linked fields of research. In this Research Topic, we see obvious and promising directions for further investigation. As we may see the rising of psychological studies of food safety and consumption in virtual contexts, the RT showed that current interests are still relatively favored phenomena in the physical world. In addition, to increase the efforts to study psychological issues of food safety and consumption in the virtual world, it might be even worthwhile if one can conduct studies to explicate the deeper factors, mechanisms, processes, etc. that drive cross- or trans-contextual phenomena between the physical and virtual worlds of food safety, governance, and consumption. For example, what would be the cognitive change in food value when one started to shift their shopping from physical to online markets? How would one's psychological state change if the experiences of shopping online vs. physically generate some conflict? What are the differences for one thing (e.g., food fraud) that simultaneously exists in both the virtual and physical food worlds? All of the logics presented behind the above-discussed articles are also applicable to the research level's concerns. For example, is there cross-level existence of one identical phenomenon (e.g., food fraud, again) in different levels of analysis with different influencing antecedent factors, theoretical mechanisms, and consequences? How do those factors, mechanisms, and outcomes at different levels influence one another? Issues similar or beyond the aforementioned may stimulate brighter imaginations for the future of psychological research in the food sector.

## Author Contributions

F-ST, X-WW, and SS contributed equally in editing this Research Topic. F-ST wrote the original draft of this article. X-WW and SS reviewed and revised it mightily. All authors contributed to the article and approved the submitted version.

## Conflict of Interest

The authors declare that the research was conducted in the absence of any commercial or financial relationships that could be construed as a potential conflict of interest.

## Publisher's Note

All claims expressed in this article are solely those of the authors and do not necessarily represent those of their affiliated organizations, or those of the publisher, the editors and the reviewers. Any product that may be evaluated in this article, or claim that may be made by its manufacturer, is not guaranteed or endorsed by the publisher.

